# An effective technique for developing the graphical polynomials of certain molecular graphs

**DOI:** 10.1038/s41598-023-31623-7

**Published:** 2023-03-23

**Authors:** Ibtisam Masmali, Muhammad Nadeem, Awais Yousaf, Ali Akbar, Abdul Razaq, Asima Razzaque

**Affiliations:** 1 Department of Mathematics, College of Science, Jazan University, 45142 Jazan, Saudi Arabia; 2 Department of Mathematics, The Islamia University of Bahawalpur, Bahawalpur, 63100 Pakistan; 3 Department of Mathematical Sciences, National College of Business Administration and Economics Sub Campus Bahawalpur, Bahawalpur, Pakistan; 4 Department of Mathematics, Division of Science and Technology, University of Education, Lahore, 54770 Pakistan; 5 Department of Basic Sciences, Deanship of Preparatory Year, King Faisal University Al Ahsa, Al Hofuf, Saudi Arabia

**Keywords:** Chemistry, Mathematics and computing

## Abstract

Counting Polynomial is the mathematical function that was initially introduced for application in chemistry in 1936 by G. Polya. Partitioning of graphs can be seen in the coefficients of these mathematical functions, which also reveal the frequency with which these partitions happen. We developed a novel and efficient method for constructing the necessary counting polynomials for a zigzag-edge coronoid formed by the fusion of a Starphene graph and a Kekulenes graph. The study's methods expand our knowledge, and its findings potentially provide insight on the topology of these chemical structures.

## Introduction

.Graph theory has several applications beyond the chemistry, including the study of chemical compounds, which are often represented as graphs. The chemical compound's graph can be represented by a molecular descriptor, a matrix, or a graph polynomial. Using the applications of Chemical Graph Theory, we reveal that the graph polynomials, molecular descriptors, and sequences associated to molecular descriptors for 2D-molecular graphs have significant implications for the domains of mathematical chemistry, quantum chemistry, QSAR, and QSPR. Moreover the Chemical graph theory or Molecular topology is a branch of Graph theory applied in the study of molecular structures represents an interdisciplinary science. Topological characterization of chemical structures allows the classification and modelling of structures with desired properties. The counting polynomials show the partition of graphical structures such as, the Omega and Theta polynomials, which count the equidistance edges, whereas Sadhana and PI polynomials count the non-equidistance edges that are discussed in the point raised in the first question. Mathematical chemistry is a field of study concerned with innovative applications of mathematics to chemistry. It is primarily concerned with the mathematical modelling of chemical reactions. Mathematical chemistry is also known as computer chemistry; however, it is not to be confused with computational chemistry. Chemical graph theory, which deals with topology such as the mathematical study of isomerism and the development of topological descriptors or indices that find application in quantitative structure–property relationships; and chemical aspects of group theory, which finds applications in stereochemistry and quantum chemistry, are major areas of research in mathematical chemistry. In mathematical chemistry the division of the graph (chemical compound) is shown by counting polynomials, and molecular descriptors are generated using these polynomials. The varied features of the chemical compound are shown by mathematical modelling of the molecular descriptors. We can grasp the structural properties of a chemical compound because it comprises graph polynomials such as counting polynomials, matching polynomials, and differential polynomials. Topological indices related to counting polynomials have been extensively investigated in mathematical chemistry via techniques of graph theory, and they have numerous applications in chemistry, especially in quantum chemistry. Please refer to^1–7^ for any more information that you may require. In recent years, numerous researchers have focused on formula construction and counting polynomials to construct molecular descriptions of various families of molecular graphs. In the field of chemical graph theory, Diudea's research concentrated on counting polynomials and the associated indices for a variety of distinct chemical structures, and he made significant contributions to chemical graph theory, See Refs.^2,3,8^. In this study, we provide a new method that is both effective and time saving for the construction of counting polynomials. Moreover, we established the Omega, Sadhana, Theta, and PI polynomials of the Zigzag-edge coronoid that was fused by the Starphene graph and the Kekulenes graph.

Let $G(V, E)$ be a graph having no isolated vertex and no loop, here $V, E$ are the vertex and edge set respectively. The number of edges between two vertices of the graph is term as the distance between any two vertices ${v}_{1}$, ${v}_{2}$ of the graph $G$ is defined as the smallest path between ${v}_{1}$, ${v}_{2}$ and it is represented by $d({v}_{1}, {v}_{2})$. If distance between vertices ${v}_{1}, {v}_{2}$ and ${v}_{3}, {v}_{4}$ are equal then the edges connected by ${v}_{1}, {v}_{2}$ and ${v}_{3}, {v}_{4}$ are called co-distance edges and mathematically if $e$ and $f$ are the edges in a graph $G$ such that $e ={v}_{1}{v}_{2}$, $f={v}_{3}{v}_{4}$ are co-distance edges, then $d({v}_{1}, {v}_{2})= d({v}_{3}, {v}_{4})$ and $d({v}_{1}, {v}_{2})= d({v}_{3}, {v}_{4})= d({v}_{1}, {v}_{4})+1= d({v}_{3}, {v}_{2})+1$. It is denoted by ‘$e co f$’, here the co-distance relation between the edges is symmetric and reflexive. Let $C(e) = \left\{f\in E\left(G\right);f co e\right\}$ be the collection of all the co-distance edges and holds the transitive property is said to be the orthogonal cut, it is denoted by $co$ of $G$. In a graph $G$ the edges which are opposite to each other in same ring or in same face form the edge strip and is called an opposite edge strip, it is denoted by ‘$ops$’and the number of edges in a strip is called length of the strip. The set of opposite edges is also called the $\mathrm{quasi}-\mathrm{orthogonal}$ cut, denoted by ‘$\mathrm{qoc}$’ here $qoc$ defined in the whole graph $G$, while the ‘$ops$’ is defined in the same face or ring. By $m(G, c)$, we mean the number of strips of length $c$. Duidea established the Omega Polynomial by using the strip length and number of strips that has the relation $\Omega (G, x)=\sum_{c}m(G, c){x}^{c}$, where $c$ is the number of edges in the strip and ‘$m(G, c)$’ is the cardinality of the strips. For more information see Refs.^9,10^. M. Ghorbani constructed the Sadhana polynomial as, $Sd(G, x) = \sum_{c}m(G, c){x}^{\left|E(G)\right|-c}$ in Ref.^11^, here, $\left|E(G)\right|$ is the order of the edge set of the graph *G*. The Theta polynomial of the graph $G$ is denoted by ‘${\varvec{\theta}}\left(G, x\right)$’ it is given by, ${\varvec{\theta}}\left(G, x\right)=\sum_{c}m\left(G, c\right)c{x}^{c}$ in Ref.^12^. In Ref.^13^, the PI polynomial is constructed as, $\pi (G, x)$ =$\sum_{c}m\left(G, c\right)c{x}^{\left|E(G)\right|-c}$.

The methods introduced by Duidea, M. Ghorbani, and A. R. Ashrafi to construct four counting polynomials have been discussed above but the method used in this study is a first-hand method and easy to construct, the Omega polynomials, Theta, Sadhana, and PI polynomials by utilizing the star expression ${\varvec{S}}{\varvec{t}}\left(G,x\right)={x}^{\left|E(G)\right|-2c}$, which is based on the size of the graph and the length of the strips. The groundbreaking method for the creation of four counting polynomials applied in this manuscript is introduced by Awais Yousaf and his colleagues to build these polynomials, provided in Ref.^14^ as follows.

### Theorem 1.1

^14^ *Let* $G$ *be* *a* *planar* *graph* *and* $\Omega \left(G, x\right)$ *be* *the* *Omega* *polynomial* *related* *to* *G,* *then*

i. $${\varvec{\theta}}\left(G, x\right){=x\Omega^{'}}\left(G, x\right)$$,
ii. $$Sd\left(G, x\right)={\varvec{S}}{\varvec{t}}\left(G,x\right)\Omega \left(G, x\right)$$,
iii. $$\pi \left(G, x\right)=x{\Omega^{'} }\left(G, x\right){\varvec{S}}{\varvec{t}}\left(G,x\right)$$.

## Zigzag-edge coronoid fused by Starphene (ZCS)

**Figure 1:**
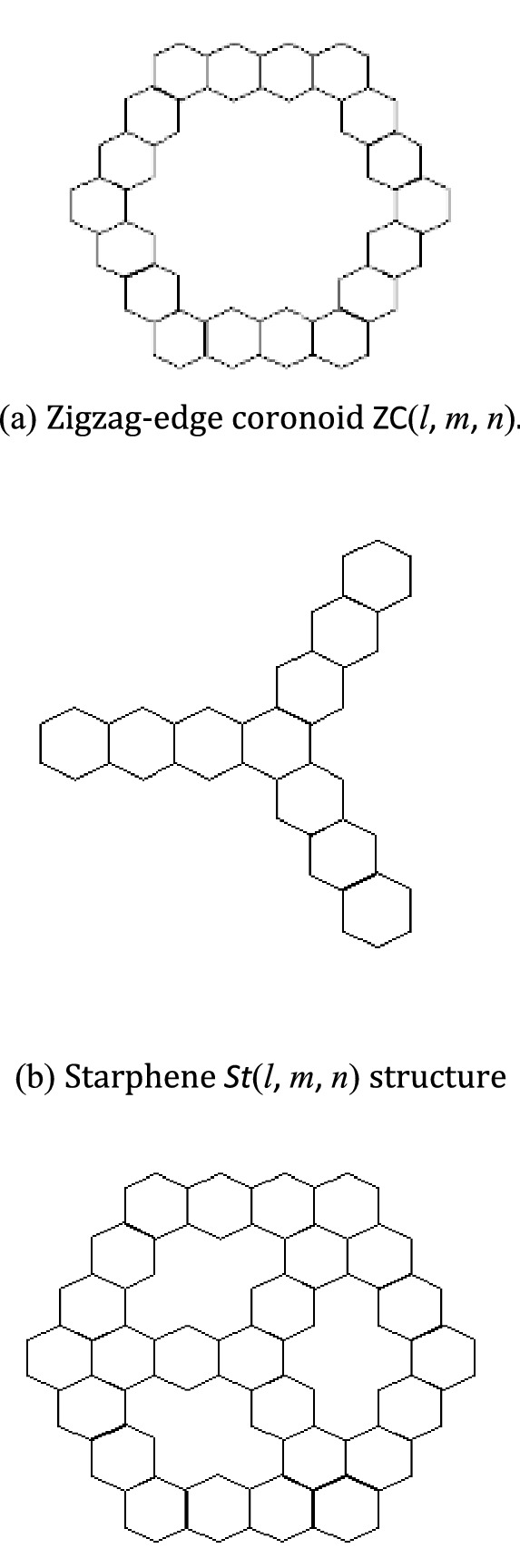
Zigzag-edge coronoid fused by Starphene $ZCS(\mathrm{4,4},4).$

The chemical compound benzene has the molecular formula ${C}_{6}{H}_{6}$ and is classified as an organic chemical. It is used as a solvent in a wide range of commercial, scientific, and industrial applications. Benzoene is a key component of gasoline and is also found in crude oil. A zigzag-edge coronoid is combined with a Starphene to form a composite benzenoid. Figure 1 shows a zigzag-edge coronoid fused by Starphene graph, which is a composite benzenoid constructed by fusing a zigzag-edge coronoid with a Starphene. The zigzag-edge coronoids ZC(*l*, *m*, *n*), shown in Fig. 1a, can be considered as a structure obtained by fusing six segments of linear polyacenes into closed loop. A starphene St(*l*, *m*, *n*), shown in Fig. 1b, is a structure obtained by fusing three linear polyacenes of length $l, m, n.$

### Theorem 2.1

*The* *Omega* *polynomial* *for* $ZCS(l, m,n)$ *graph* *for*$, m,n \ge 3$*,* *is;*

$$\Omega \left(G, x\right)= 9{x}^{n+1}+ \left(12n-27\right){x}^{2} + 6{x}^{3}.$$

### Proof

Since Omega polynomial of a graph is defined as:

$$\Omega \left(G, x\right)= \sum_{c}m\left(G, c\right){x}^{c},$$

where “c” is the length of strip. By using the values from Table 1 and substituting in above relation, we get the required polynomial for $ZCS(l, m,n)$ graph as follows;

**Table 1:** Edge partition of $ZCS(l, m,n)$.

Type of strip	Length of strip ‘c’	Cardinality of strips of length ‘c’ (qoc)
${s}_{1}$	$n+1$	$9$
${s}_{2}$	$2$	$12n-27$
${s}_{3}$	$3$	$6$

$$\Omega \left(G, x\right)= 9{x}^{n+1} + \left(12n-27\right){x}^{2}+ 6{x}^{3}.$$

### Theorem 2.2

*Let* *Ω* *(*$G, x$*)* *be* *the* *Omega* *Polynomial* *for* $ZCS(l,m,n)$ *graph* $G$ *then* *Theta,* *Sadhana* *and* *PI* *polynomial* *of* $G$ *are*

i. $$\theta (G, x) = 9(n+1){x}^{n+1} + 2(12n-27){x}^{2}+18{x}^{3}$$
ii. $$Sd(G, x) = 9{x}^{32n-28} + (12n-27){x}^{33n-29} + 6{x}^{33n-30}$$
iii. $$\pi \left(G,x\right) = 9\left(n+1\right){x}^{32n-28} + 2\left(12n-27\right){x}^{33n-29} + 18{x}^{33n-30}.$$

### Proof

(i) From Theorem 2.1 the Ω ($G, x$) for graph shown in Fig. 1 is
  $$\Omega \left(G, x\right)= 9{x}^{n+1} + \left(12n-27\right){x}^{2}+ 6{x}^{3}.$$
  Then by using Theorem 1.1$\theta (G, x)$ is given by
  $$\theta \left(G, x\right)= x\Omega {^{\prime}}\left(G, x\right).$$
  Putting values in the above relation we get
  $$x\Omega {^{\prime}}\left(G, x\right)=9\left(n+1\right){x}^{n+1} + 2\left(12n-27\right){x}^{2}+18{x}^{3}.$$
  Hence the required $\theta (G, x)$ for $ZCS(l,m,n)$ graph is:
  $$\theta \left(G, x\right)=9\left(n+1\right){x}^{n+1} + 2\left(12n-27\right){x}^{2}+18{x}^{3}.$$
(ii) In Theorem 1.1, $Sd(G, x) =\sum_{c}{x}^{|E(G)| - 2c} \Omega (G,x)$
  The size of the Zigzag-edge coronoid fused by Starphene graph is $|E(G)| = 33(n-3) + 72$ and the star expression is ${x}^{33\left(n-3\right)+ 72-2c}$ and c is same as already defined. Using these values in above equation and simplifying, we get,
  $$Sd\left(G, x\right)= 9{x}^{32n-28} + \left(12n-27\right){x}^{33n-29} + 6{x}^{33n-30}.$$
(iii) The PI polynomial is defined in Theorem 1.1 as under;
  $$\pi \left(G,x\right) = \sum_{c}{x}^{\left|E\left(G\right)\right|-2c+1} \Omega {^{\prime}}\left(G,x\right).$$
  Substituting the value of $|E(G)| = 33(n-3) + 72$ in the above equation and simplifying, we obtained $\pi \left(G,x\right)$ as:
  $$\pi \left(G,x\right) = 9\left(n+1\right){x}^{32n-28} + 2\left(12n-27\right){x}^{33n-29} + 18{x}^{33n-30}.$$

## Kekulenes graph

The kekulean and non-kekulean structures in benzenoids have important properties with respect to chemical point of view. Mostly, it is known that benzenoids having different numbers of starred and unstarred vertices contains no kekulean structure. They have color excess and are referred to as non-kekulean benzenoids. Similarly, the benzenoid having equal number of starred and unstarred vertices necessarily possess kekulean structure. According to Gutman, the equality of starred and unstarred vertices is necessary and sufficient condition for a benzenoid structure to be Kekulean. However, it is not true, non-Kekulean with equal number of starred and unstarred vertices were detected relatively early, and were letter referred to as concealed non-Kekulean$.$ It has been demonstrated that there exist exactly eight systems of this category. If we eliminate the edge-cut, then the graph is decomposed into two parts and is baptized as non-Kekulean structure of Benzenoid graph. Kekulenes graph has drawn greater interest from academics in recent years because of its electronic structure. The synthesis and categorization of these compounds make it clear that, despite their p electrons' delocalization in an annulenoid pattern, these molecules' individual form benzoid-type rings. The partition of Kekulenes graph is presented in Table 2.

**Table 2:** Partition of Kekulenes graph.

Type of strip	Length of strip ‘c’	Cardinality of strips of length ‘c’ (qoc)
${s}_{1}$	$n+1$	$6$
${s}_{2}$	$n+2$	$6$
${s}_{3}$	$3$	$12n-30$

### Theorem 3.1

*The* *Omega* *polynomial* *for* *Kekulenes* *graph* *is:*

$$\Omega \left(G, x\right)= 6{x}^{n+1}+ 6{x}^{n+2} + \left(12n-30\right){x}^{3}.$$

### Proof

Since Omega polynomial of a graph is defined as:

$$\Omega \left(G, x\right)= \sum_{c}m\left(G, c\right){x}^{c},$$

where “c” is the length of strip. By substituting values in above relation and simplifying, we get the required Omega polynomial for Kekulenes graph as follows:

$$\Omega \left(G, x\right)= 6{x}^{n+1}+ 6{x}^{n+2} + \left(12n-30\right){x}^{3}.$$

### Theorem 3.2

*Let* *Ω(*$G, x$*)* *be* *the* *Omega* *polynomial* *for* *Kekulenes* *graph* $G$ *then,* *Theta,* *Sadhana,* *and* *PI* *polynomial* *of* $G$ *are:*

i. $$\theta \left(G, x\right)= 6\left(n+1\right){x}^{n+1}+ 6\left(n+2\right){x}^{n+2}+ 3\left(12n-30\right){x}^{3}$$
ii. $$Sd(G, x) = 6{x}^{47n-73} + 6{x}^{47n-74} + (12n-30){x}^{48n-75}$$
iii. $$\pi (G,x) = 6(n+1){x}^{47n-73} + 6(n+2){x}^{47n-74}+ 3(12n-30){x}^{48n-75}$$

### Proof

**Figure 2:**
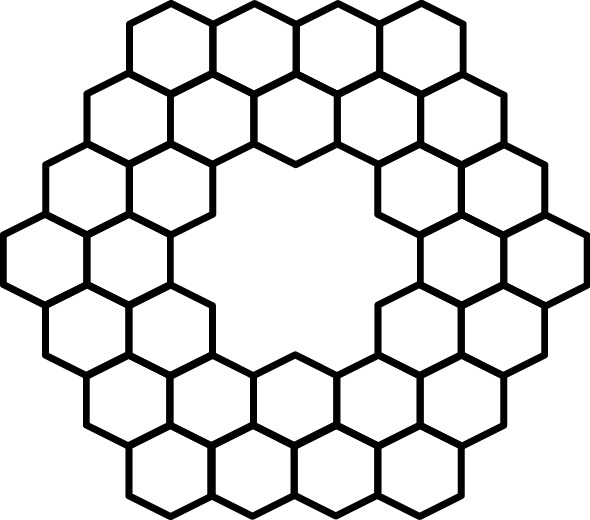
Kekulenes graph.

(i) As Omega polynomial of Kekulenes graph shown in Fig. 2 is:

  $$\Omega \left(G, x\right)= 6{x}^{n+1}+ 6{x}^{n+2} + \left(12n-30\right){x}^{3}.$$
  Then by using Theorem 1.1$\theta (G, x)$ is given by;
  $$\theta \left(G, x\right)= x\Omega {^{\prime}}\left(G, x\right).$$
  Putting values in the above relation we get
  $$x\Omega {^{\prime}}\left(G, x\right)= 6\left(n+1\right){x}^{n+1}+ 6\left(n+2\right){x}^{n+2} + 3\left(12n-30\right){x}^{3}.$$
  Hence theta polynomial of Kekulenes graph is;
  $$\theta \left(G, x\right)= 6\left(n+1\right){x}^{n+1}+ 6\left(n+2\right){x}^{n+2}+ 3\left(12n-30\right){x}^{3}.$$
(ii) Using Theorem 1.1$Sd(G, x) =\sum_{c}{x}^{|E(G)| - 2c} \Omega (G,x)$
  The size of the Kekulenesgraph is $|E(G)| = 48(n-2) + 24$ and star expression for Kekulenes is ${x}^{48\left(n-2\right)+ 24-2c}$. Using these values in the above relation and simplifying, we get,
  $$Sd\left(G, x\right)= 6{x}^{47n-73} + 6{x}^{47n-74} + \left(12n-30\right){x}^{48n-75}.$$
(iii) PI polynomial is defined by Awais Yousaf and his fellow is as under^14^.
  $$\pi \left(G,x\right) = \sum_{c}{x}^{\left|E\left(G\right)\right|-2c+1} \Omega {^{\prime}}\left(G,x\right).$$
  Substituting the value of $|E(G)| = 48(n-2) + 24$ and c already defined also putting the values of the derivative of Omega Polynomial and simplifying, we obtained PI polynomial for Kekulene graph as:
  $$\pi \left(G,x\right) = 6\left(n+1\right){x}^{47n-73} + 6\left(n+2\right){x}^{47n-74}+ 3\left(12n-30\right){x}^{48n-75}.$$

## Conclusion

The study presented an innovative approach that significantly contributes to the existing body of knowledge in mathematical chemistry and chemical graph theory. The novel method involves the generation of Theta, PI, and Sadhana polynomials by utilizing an Omega polynomial for a given graph. The derived polynomials are useful for computing the Theta, PI, and Sadhana indices without the need to generate these polynomials explicitly. The Theta, PI, and Sadhana indices are crucial parameters in mathematical chemistry and chemical graph theory, which describe the structural properties of molecules. However, the computation of these indices can be computationally intensive and time-consuming, especially for large graphs. The approach presented in this study provides an efficient way to calculate these indices, thereby saving computational time and resources. The innovative method involves utilizing an Omega polynomial, which is a well-established polynomial in chemical graph theory that encodes structural information about a given graph. By manipulating the Omega polynomial, the Theta, PI, and Sadhana polynomials are generated, providing a compact representation of the structural information encoded in the Omega polynomial.

By offering a new and efficient approach for determining the Theta, PI, and Sadhana indices, the novel technique presented in this article contributes an important addition to the fields of mathematical chemistry and chemical graph theory. The method has the potential to impact various applications, including drug discovery, material science, and catalysis, where the characterization of molecular structures is crucial.

## Data Availability

All data generated or analysed during this study are included in this published article.
